# Spectral Selectivity of Plasmonic Interactions between Individual Up-Converting Nanocrystals and Spherical Gold Nanoparticles

**DOI:** 10.3390/ma10080905

**Published:** 2017-08-04

**Authors:** Dawid Piątkowski, Mikołaj K. Schmidt, Magdalena Twardowska, Marcin Nyk, Javier Aizpurua, Sebastian Maćkowski

**Affiliations:** 1 Institute of Physics, Faculty of Physics, Astronomy and Informatics, Nicolaus Copernicus University, Grudziadzka 5, 87-100 Torun, Poland; magda@fizyka.umk.pl (M.T.); mackowski@fizyka.umk.pl (S.M.); 2 Centro de Física de Materiales (MPC, CSIC-UPV/EHU) and Donostia International Physics Center (DIPC), Paseo Manuel de Lardizabal 5, 20018 Donostia-San Sebastián, Spain; mikolaj.schmidt@mq.edu.au (M.K.S.); aizpurua@ehu.eus (J.A.); 3 Advanced Materials Engineering and Modelling Group, Faculty of Chemistry, Wrocław University of Science and Technology, Wybrzeże Wyspiańskiego 27, 50-370 Wroclaw, Poland; marcin.nyk@pwr.edu.pl

**Keywords:** plasmonics, up-conversion, confocal microscopy

## Abstract

We experimentally demonstrate strong spectral selectivity of plasmonic interaction that occurs between α-NaYF_4_:Er^3+^/Yb^3+^ nanocrystals, which feature two emission bands, and spherical gold nanoparticles, with plasmon frequency resonant with one of the emission bands. Spatially–resolved luminescence intensity maps acquired for individual nanocrystals, together with microsecond luminescence lifetime images, show two qualitatively different effects that result from the coupling between plasmon excitations in metallic nanoparticles and emitting states of the nanocrystals. On the one hand, we observe nanocrystals, whose emission intensity is strongly enhanced for both resonant and non-resonant bands with respect to the plasmon resonance. Importantly, this increase is accompanied with shortening of luminescence decays times. In contrast, a significant number of nanocrystals exhibits almost complete quenching of the emission resonant with the plasmon resonance of gold nanoparticles. Theoretical analysis indicates that such an effect can occur for emitters placed at distances of about 5 nm from gold nanoparticles. While under these conditions, both transitions experience significant increases of the radiative emission rates due to the Purcell effect, the non-radiative energy transfer between resonant bands results in strong quenching, which in that situation nullifies the enhancement.

## 1. Introduction

An emitter localized close to a metallic nanoparticle can experience modification of its absorption ($\gamma_{\mathrm{abs}}$), emission ($\gamma_{r}$) and non-radiative ($\gamma_{\mathrm{nr}}$) transition rates. The net result of this interaction, which depends predominantly on the distance between the emitter and the metallic nanoparticle, as well as on their relative spectral properties [1,2,3,4,5,6], can be enhancement or quenching of the emission intensity. Furthermore, carefully engineered hybrid nanosystems can also reach the so-called strong coupling regime, manifesting itself by the modification of both the extinction [7] and emission spectra of an emitter [8].

The distance dependence of luminescence enhancement has been demonstrated using a near-field optical microscope, where either a gold tip or single metallic nanoparticle attached to the AFM tip were used [2,3,4]. The measurements have shown that the optimal distance between an emitter and a subwavelength plasmonic nanoparticle for luminescence enhancement is about 10–15 nm [5]. For smaller distances, non-radiative energy transfer between the emitter and metallic nanoparticle starts to dominate, which leads to strong quenching of the emission. In such geometries, however, the spectral properties of the gold tip cannot be easily tuned in order to ensure optimal spectral matching with absorption or emission bands of the studied emitters.

Of particular interest is the research devoted to understand the influence of the spectral properties of the plasmon resonance (position and shape) on the optical properties of nearby emitters [6]. Besides silver nanowires featuring quite broad resonance [9], smaller nanoparticles like nanorods [10], nanotriangles [11] or nanostars [12,13], demonstrate narrower resonance peaks, whose position can be controlled by the particle morphology. Thus, the most suitable nanoparticles to develop insightful spectroscopic investigations seem to be those showing a single, relatively narrow resonance band, selectively matching the emission lines of the considered luminescence probe.

Previous investigations demonstrated a general rule that the largest values of the luminescence enhancement factors are typically observed in configurations where emission is spectrally red-shifted with respect to the plasmon resonance, i.e., where the absorption of the metallic nanoparticle is significantly smaller [14]. An open question remains regarding the nature of the interaction between a narrow plasmon resonance and a multimodal emitter that features several narrow, independent emission bands.

In this paper we investigate the optical properties of a hybrid structure composed of spherical gold nanoparticles and nanocrystals with bimodal emission: one emission band centered at 540 nm which is resonant with the plasmon resonance of gold nanoparticles, and another emission band centered at 650 nm which is strongly red-shifted with respect to the plasmon resonance. By probing the interaction at both wavelengths we are able to find nanocrystals exhibiting almost complete quenching of 540 nm emission, while at the same time emission at 650 nm is enhanced. In accordance with theoretical modeling of quantum yields, we ascribe this behavior to nanocrystals that are placed at short distances from gold nanoparticles. Theoretical calculations performed for emitter-metal nanoparticle separation distance of 5 nm show, that radiative transition rates of both emissions are significantly enhanced. The green emission, however, shows strong decrease of the luminescence intensity due to nonradiative energy transfer to the nearby metallic nanoparticles.

## 2. Materials and Methods

**Figure 1:**
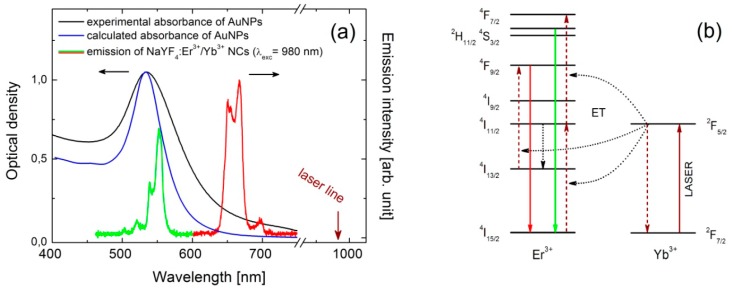
Experimental (black curve) and theoretical (blue curve) absorbance spectra of the colloidal AuNPs together with emission spectrum of the single NC (green/red curve) under 980 nm laser diode excitation (**a**). Energy diagram of the up-conversion transitions observed in the Er^3+^/Yb^3+^ system (**b**).

For experimental studies we used α-NaYF_4_ nanocrystals (NCs) doped with Er^3+^ (2 mol %) and Yb^3+^ (20 mol %) ions [15]. NCs were synthesized via a thermal decomposition reaction of trifluoroacetate precursor in mixture of technical grade chemicals, octadecene and the coordinating ligand, oleic acid. In a typical synthesis fixed amounts of rare-earth oxides were mixed with ~50% aqueous trifluoroacetic acid and heated to ~90 °C to obtain lanthanide trifluoroacetate precursors. The residual solvents were evaporated to dryness. Next, the obtained trifluoroacetate precursor salt was added to flask with octadecene, oleic acid and sodium trifluoroacetate. The solution was then heated to 100 °C under vacuum with stirring for 30 min for total oxygen and water removal, during which the flask was purged with dry nitrogen every 5 min. The resulting solution was then heated to 300 °C in nitrogen atmosphere and kept under vigorous stirring for 1 h. After synthesis, the mixture was cooled down to room temperature and precipitated by the use of methanol and acetone mixture (1:1 by volume) and collected by centrifugation at 10,000 rpm for 10 min. Finally, nanocrystals with an average diameter of 30 nm were dispersed in chloroform, and formed an optically stable in time colloid. Spectroscopically, erbium and ytterbium doped NCs manifest strong anti-Stokes luminescence under near infrared excitation, commonly referred to as energy transfer up-conversion (ETU) [16]. Excitation at 980 nm activates two emission bands in the visible spectral range, at 540 nm and 650 nm, which can be assigned to the ^2^H_11/2_ + ^4^S_3/2_ → ^4^I_15/2_ and ^4^F_9/2_ → ^4^I_15/2_ transitions in Er^3+^ ions, respectively (Figure 1a,b) [17].

Gold nanoparticles (NPs) were synthesized using the method of chemical reduction of the gold precursor in boiling water [18]. Chloruric acid solution (1 mM, 20 mL) was heated to the boil in a round-bottom flask, at the time trisodium citrate water solution (38.8 mM, 2 mL) was added dropwise. After 15 min of heating and vigorous stirring the mixture was cooled down to the room temperature. Obtained nanoparticles feature an average diameter of about 30 nm and are optically active in the visible range, with strong plasmon resonance centered at 530 nm, as shown in Figure 1a. Thereby, the green emission band of erbium ions (540 nm) is resonant with the plasmon resonance, while the red emission band of Er^3+^ (650 nm) is essentially off-resonant. In this way, we can probe wavelength-sensitive interactions between NCs and AuNPs.

The samples were prepared as follows. First, we prepared a reference sample containing only α-NaYF_4_:Er^3+^/Yb^3+^ nanocrystals, by spin-coating 20 μL of low concentrated solution of the NCs (0.5 mg/mL) on a glass coverslip. The hybrid nanostructure containing both nanocrystals and gold nanoparticles was prepared in the same way, but in this case a highly concentrated solution of gold nanoparticles was deposited on a coverslip just before we spin-coated the nanocrystal solution. Thus, we used neither polymer nor any other spacer stabilizing or separating particles [14], which should assure close proximity between both components of the structure.

The optical properties of the structures were investigated by a confocal fluorescence microscope (Eclipse Ti-S, Nikon, Amsterdam, The Netherlands), equipped with a high numerical aperture oil-immersion objective (Plan Apo 60× NA = 1.4, Nikon, Amsterdam, The Netherlands). For excitation we used a fiber-coupled single-mode laser diode (980 nm) operating in both continuous-wave (CW) and pulse modes. An unpolarized CW excitation beam was used for mapping of the luminescence intensity and 1 μs long pulses were generated in order to perform time-resolved experiments. The up-conversion luminescence signal was detected by an avalanche photodiode (APD) (SPCM-16, PerkinElmer, Vaudreuil, Canada) through band-pass filters (540/40 nm or 650/30 nm) for green and red emission bands of Er^3+^, respectively. The samples were mounted on a piezoelectric stage, synchronized with read-out of SPCM for raster scanning. Spatial resolution of the optical system was diffraction-limited at approximately 450 nm. For time-resolved experiments we connected the APD to a multiscaler card (MSA-300, Becker&Hickl, Berlin, Germany). Luminescence transients were collected with 1 µs resolution in a time window of 0–1.5 ms. The card and the laser were triggered and synchronized by a programmable pulse generator (Keithley 3390, Solon, OH, USA).

## 3. Results

**Figure 2:**
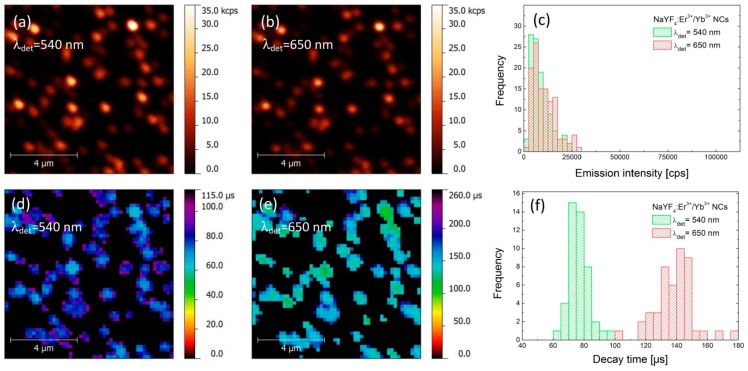
Photoluminescence intensity maps of α-NaYF_4_:Er^3+^/Yb^3+^ nanocrystals (reference sample) observed at 540 nm (**a**) and 650 nm (**b**), together with statistical distributions of luminescence intensities (**c**). FLIM images of green and red emissions are presented in sections (**d**,**e**), respectively, together with statistical distributions of decay times shown in section (**f**).

First, we carried out a photoluminescence (PL) imaging experiment for a reference structure, where α-NaYF_4_:Er^3+^/Yb^3+^ nanocrystals were deposited on a glass coverslip at a concentration that allowed for probing the emission properties of individual nanocrystals. The spatial distribution of the PL intensity for both green (540 nm) and red (650 nm) emission bands, acquired over the same area of the sample, is shown in Figure 2a,b, respectively. The excitation wavelength used in the experiment was 980 nm. Both maps feature randomly distributed spots, which we attribute to the emission from individual or few close-lying α-NaYF_4_:Er^3+^/Yb^3+^ nanocrystals [3]. Importantly, the spatial positions of the nanocrystals observed for both emission bands are identical, which indicates that all of the emission observed in the experiment is associated with the up-conversion in the nanocrystals. In order to extract the emission intensities for both emission bands we determined the maximum intensities for approximately 100 spots. The result is presented as histograms in Figure 2c. We note that emission intensities of both green and red emissions are comparable, reaching up to 25–30 kcps (kilo-counts per second), with average values of about 10 kcps.

As the interactions with plasmon excitations in metallic nanoparticles frequently yield strong modifications of the radiative properties of nearby emitters, in order to quantify the observed effects, it is necessary to measure—in addition to PL intensity—also the PL dynamics, especially the FLIM (Fluorescence-lifetime Imaging Microscopy) maps. Thus, we have carried out spatially-resolved PL lifetime imaging experiment with microsecond temporal resolution [19,20]. Briefly, we raster scanned the sample and measured the luminescence transient for each position on the piezo stage for both emissions. Subsequently, we estimated the intensity-weighted average lifetime and assigned the obtained value to each pixel on the map [19]. The resulting spatial distributions of the PL decay time obtained for the same regions as shown in Figure 2a,b, are displayed in Figure 2d,e. Some reduction in resolution observed for FLIM maps is due to the increase in the pixel size, introduced in order to reduce the acquisition time of the whole map. First of all, we observe perfect spatial correspondence between the positions of the NCs, regardless of the chosen emission band or the mode of the experiment (PL or PL dynamics). The images of PL lifetimes feature quite narrow distributions for both emission bands, with average values of the decay time of about 75 μs for 540 nm and 140 μs for 650 nm emission, respectively. The broadening of the lifetime distributions (Figure 2f) can be attributed to the nanocrystals size (surface to volume ratio) variations and differences in interaction between Er^3+^ ions and surface ligands [21]. The obtained values are typical for such nanocrystals [19].

**Figure 3:**
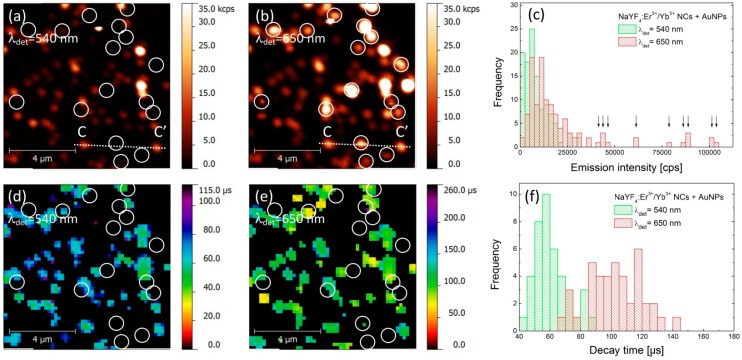
Photoluminescence intensity maps of α-NaYF_4_:Er^3+^/Yb^3+^ nanocrystals deposited on gold nanospheres observed at 540 nm (**a**) and 650 nm (**b**). Circles indicate position of the NCs which are quenched in the green channel but remain active in the red detection channel. Statistical distributions of the luminescence intensities are presented in section (**c**), where arrows denote emission from the NCs quenched in the green channel. FLIM images of green and red emissions are presents in sections (**d**,**e**), respectively, together with statistical distributions of decay times in section (**f**).

An analogous experiment has been carried out for the hybrid nanostructure, where the up-converting NCs were deposited on gold nanoparticles. PL intensity maps collected for green and red emission bands are presented in Figure 3a,b, respectively. As both images were acquired for the same sample area, it is possible to find a generally similar pattern of emission spots. However, the correspondence between the two images is not perfect, as was the case for the reference sample. Indeed, the PL maps are less homogeneous and contain many bright spots, which are apparent in particular for the 650 nm emission. Furthermore, there is a subset of nanocrystals where there is no spatial correspondence between intensities measured for green and red emission bands. These are marked in Figure 3a,b with circles, and indicate nanocrystals which feature very strong emission at 650 nm and essentially no emission at 540 nm. Consequently, we can distinguish two qualitatively different behaviors of the structure, where the NCs are deposited on AuNPs.

**Figure 4:**
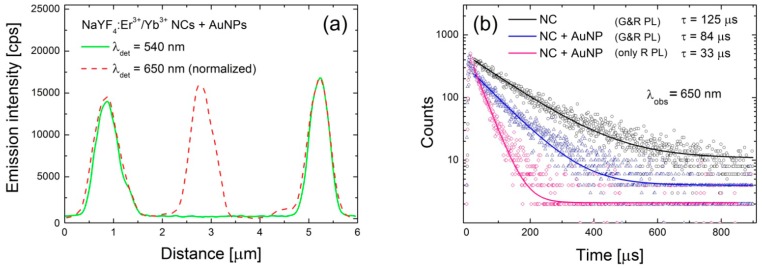
Emission intensity cross-section plotted over CC’ line in Figure 3a,b, acquired for green and red emissions (**a**). Luminescence decay curves of red emission measured for: reference NCs (black line), NCs coupled with AuNPs featuring both emissions (blue line) and NCs coupled with AuNPs featuring red emission and completely quenched green emission (pink line) (**b**).

In order to get insight into the effects responsible for such dramatic changes, we analyzed the data in a similar way as for the reference structure. The result is shown in Figure 3c, where histograms of PL intensity measured for both green and red emission bands are plotted. With respect to the reference sample, we observe a significant shift of the average emission intensity at 650 nm, from 10 kcps to about 20 kcps (Figure 3c). The intensities extracted for the nanocrystals that feature no emission in the 540 nm band are marked with arrows in Figure 3c. In the case of the green emission the change of the average PL intensity upon coupling with AuNPs is rather minute, although it seems like there are more NCs with emission intensities below 3 kcps. An example of intensity profile extracted along the lines (CC’) shown in Figure 3a,b is plotted in Figure 4a showing complete quenching of the green emission of the nanocrystal placed in the middle.

The results of spatially-resolved PL lifetime imaging are shown in Figure 3d,e for 540 nm and 650 nm band, respectively. Similarly to the case of PL intensity, the decay time maps are also less homogeneous that those of the reference sample. Furthermore, we can also distinguish two subsets of nanocrystals: for most of the nanocrystals we can estimate the average PL decay time for 540 nm and 650 nm. Remarkably for these nanocrystals, the values of PL decay times are substantially shorter for both emissions and are equal—on average—to 60 μs and 100 μs for green and red emission bands, respectively (Figure 3f). The shortening of PL lifetime proves the interaction between the nanocrystals and plasmon excitations in AuNPs that results in an increase of the spontaneous decay rate. This effect is present for both emission bands, although the enhancement of the green emission is less (20% reduction of the lifetime) than that of the red (28% reduction of the lifetime). The second set of nanocrystals is marked with circles in Figure 3d,e, and these are the same nanocrystals that feature essentially no emission at 540 nm. In this case, we frequently find no signal in the PL lifetime images for either emission band. The absence of some of the values of PL lifetime stems from limitations of the experimental technique. On the one hand, in order to determine the PL lifetime, certain intensity of the emission is required. Since we observe no emission of these nanocrystals in the 540 nm band, it is not possible to extract the PL decay rate. On the other hand, the shortest decay time that can be detected is equal to 30 μs. Thus, we interpret the absence of PL lifetimes in Figure 3e to the fact, that the PL decay is faster than 30 μs.

This analysis is supported by the PL transients collected for 650 nm band and shown in Figure 4b. The presented transients have been measured for three different configurations: reference NCs (125 μs, black curve), NCs deposited on the gold NPs featuring both red and green emissions (83 μs, blue curve), and finally NC deposited on the gold NPs showing only red emission and completely quenched green emission (33 μs, pink curve). As the interaction between emitters and metallic nanoparticles depends strongly on the distance between them, we tentatively attribute the qualitative changes observed experimentally to structures with different distance between NCs and AuNPs.

## 4. Calculations and Data Analysis

We interpret the experimental results presented in the previous section with a theoretical analysis that describes the NCs luminescence intensity in terms of emission rates and quantum efficiencies for a hybrid emitter-metal nanoparticle system. To calculate the modification of the emission rates and the quantum efficiencies for each of the transitions, we have modeled the interaction between single emitters and spherical metallic nanoparticles using an exact classical electrodynamical model described elsewhere [22,23,24]. Since the two relevant relaxation paths do not share a common excited state of the Er^3+^ ions, each one is described by considering a single dipolar emitter, positioned 5, 15 or 30 nm from the surface of the gold nanosphere of 30 nm in diameter, with the dielectric function of metal taken from the literature [25]. First, we calculated the absorbance spectrum of a single gold nanoparticle, immersed in a homogeneous medium of refractive index 1.4, using the Mie theory and depicted with the blue line in Figure 1 [26,27]. The absorption peak at 530 nm reproduces the experimental results. However, it should be mentioned that the experimental band is much broader, mostly due to the dispersion of the nanoparticles sizes.

**Figure 5:**
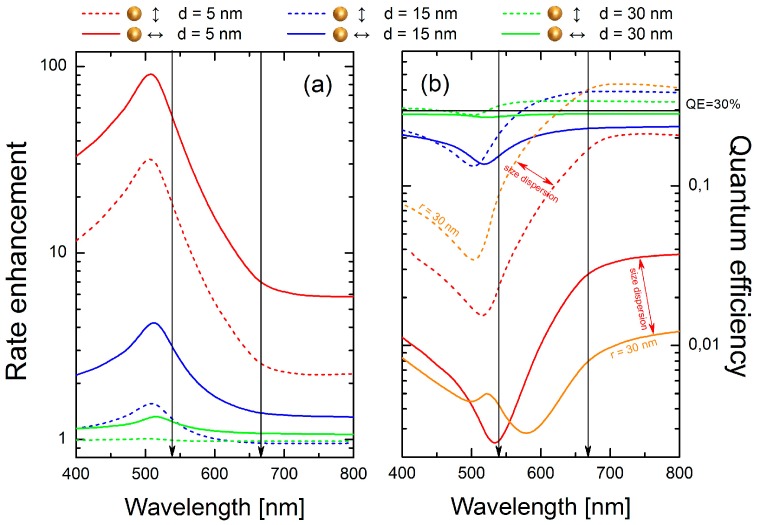
Radiative rate enhancement plotted versus wavelength, calculated for parallel (dashed lines) and perpendicularly (solid lines) oriented dipoles, at a distance d = 5 (green lines), 15 (blue lines) and 30 nm (red lines) from the surface of AuNP (**a**). Quantum efficiencies calculated for the same distances and dipole orientations are presented in (**b**). Calculations have been performed for an Au nanoparticle with radius r = 15 nm. For comparison, quantum efficiencies calculated for a bigger nanoparticle (r = 30 nm), with the emitter placed d = 5 nm from its surface are plotted in orange lines (**b**). Vertical arrows denote spectral position of the NCs emission lines.

Quantum efficiency of the emission from uncoupled nanocrystals ($QE_{0}$) was assumed to be about $QE_{0}$ = 30% for both considered emissions and have been estimated by comparing experimental and theoretical emission rates [3]. The latter have been estimated using the Judd-Ofelt approach [17,28,29]. Calculations of the modification of the spontaneous decay rate $F_{p}$ and QE from the Er^3+^ emitters, shown in Figure 5a,b, have been performed for both parallel and perpendicular orientations of emitters of the NCs with respect to the surface of the gold sphere. We note that the ions excited in a nanocrystal are modeled as permanent—not induced—dipolar emitters, as we do not consider the nonlinear excitation process. Spectra of these quantities have been corrected to account for the non-unitary intrinsic quantum efficiency of uncoupled emitters $QE_{0}$.

(1) $$F_{p}=1-QE_{0}+QE_{0}F_{p}^{0,tot}$$

and

(2) $$QE=QE_{0}\frac{F_{p}^{0,rad}}{F_{p}},$$

where $F_{p}^{0,tot}$ and $F_{p}^{0,rad}$ denote the enhancement of the total and radiative decay rate, respectively, of a dipolar emitter with unitary intrinsic quantum efficiency.

With the decay rate enhancement factors much larger than 1, the spontaneous decay rate enhancement $F_{p}$ shown in Figure 5a with dashed/solid lines for the dipoles oriented parallel/perpendicular to the surface of the sphere, is determined primarily by the last term in Equation (1). Expectedly, it grows rapidly with the decreasing distance between the emitter and the sphere, with the maximum rate enhancement ($F_{p}$ ≈ 100) achieved for d = 5 nm and the emission wavelength near the peak of the absorbance, around 510 nm. The enhancement peak extends over a broad spectral range, from about 400 nm up to 700 nm. Thus, for both emission wavelengths we expect an increase of the emission rates (shortening of the luminescence decay time), in agreement with what was observed in the experiment.

Strong competition between emission rate enhancement and luminescence quenching (absorbance of NPs) can be clearly seen in Figure 5b, where the calculated quantum efficiencies are plotted. Starting from distant dipoles (d = 30 nm, green lines), weak luminescence quenching in the range 400–500 nm for perpendicular dipoles and weak emission enhancement in the range 550–800 nm for parallel dipoles is observed. By reducing the separation distance to 15 nm (blue lines), emission enhancement for parallel dipoles is still observed in the range from 600 to 800 nm, where as for perpendicular dipoles luminescence is quenched.

An interesting situation occurs for a distance equal to 5 nm (red lines). For perpendicularly oriented dipoles (red solid line), quenching dominates in the whole spectral range, with QE reaching a minimum at 540 nm. However, for parallel dipoles we observe a more complex behavior. Whereas strong luminescence quenching from 400 to 650 nm is observed, at longer wavelengths luminescence can be either slightly quenched or even enhanced, assuming the presence of both smaller (r = 15 nm) or larger (r = 30 nm, orange lines) Au nanospheres, respectively. Indeed, size dispersion of the metallic nanoparticles manifests itself in the calculated extinction spectra (Figure 1) and should be taken into account in our analysis. Calculations performed for larger nanospheres (orange lines in Figure 5b) confirm that in the range from 550 to 650 nm QE grows from about 10% up to 40%. For this reason, nanocrystals featuring strongly quenched green emission are still observed in the red channel (Figure 4a). Such emitters feature a strongly reduced luminescence decay time, which we present in Figure 4b. It must be noted that extremely intense 650 nm emission from NCs placed close to the AuNPs cannot be explained only by radiative rate enhancement. Thus, we interpret this as being due to the contribution from an enhanced absorption process that usually takes place for dipoles placed very close to the metallic nanostructures [2]. Nonetheless, the trend of the transition from luminescence quenching to luminescence enhancement, probed by green and red emissions of Er^3+^ ions, is well described by the presented model.

As results from Figure 5a, the emission rate enhancement increases with a decrease in the distance between NCs and gold NPs decrease. This increase is more distinct for green than for red emission. However, we observed comparable relative shortenings of decay times for both emission bands, statistically about 20–30% on average (Figure 2f and Figure 3f). Nevertheless, from an experimental point of view, such values seem to be reasonable. According to Figure 5b, the quantum efficiency of green emission estimated for short distances is extremely low. Thus, nanocrystals localized very close to the gold NPs simply cannot contribute to the transients statistics.

We also note that in the particular case of rare earth ions doped materials, by introducing metallic nanoparticles, complex mechanisms of up-conversion luminescence can be tuned and easily disentangle. In the particular case of erbium activated NCs coupled with AuNPs, green emission is strongly quenched while red remains undisturbed or enhanced. This observation proves that the low energetic ^4^F_9/2_ level (red emitting) cannot be efficiently populated by non-radiative relaxation from higher ^2^H_11/2_ + ^4^S_3/2_ levels (green emitting), but with all certainty each emission is activated via completely independent excitation channels (Figure 1b). Thus, in Er^3+^/Yb^3+^ system, only the two-channels excitation mechanism of the up-conversion luminescence, discussed in this work, should be considered [16].

## 5. Conclusions

In this paper, we presented spectroscopic properties of α-NaYF_4_:Er^3+^/Yb^3+^ nanocrystals combined with gold nanospheres. We demonstrated that plasmonic interactions that take place between them are wavelength selective. By analyzing the anti-Stokes emission bands of Er^3+^ we showed that under certain conditions, red emission line can be enhanced while, at the same time, the green one can be strongly quenched. Moreover, the process depends on the emitter-particle separation distance, and intensifies for extremely close lying NCs and AuNPs. Our results have been confronted with theoretical calculations that confirmed that quenching of the green emission should be attributed to the energy transfer from NCs to gold NPs, whereas the observed red luminescence enhancement could be attributed to the enhancement of the emission rate and absorption.
